# Magnetic compression anastomosis is effective in treating stenosis after esophageal cancer surgery: a case report

**DOI:** 10.1186/s40792-020-00974-y

**Published:** 2020-08-17

**Authors:** Tetsuro Isozaki, Kentaro Murakami, Eigoro Yamanouchi, Masaya Uesato, Takeshi Toyozumi, Yoshio Koide, Soichiro Tsukamoto, Haruhito Sakata, Koichi Hayano, Masayuki Kano, Hideki Hayashi, Hisahiro Matsubara

**Affiliations:** 1 Department of Frontier Surgery Graduate School of Medicine, Chiba University, 1-8-1 Inohana, Chuo-ku, Chiba-shi, Chiba, 260-8670 Japan; 2 Department of Radiology, International University of Health and Welfare Hospital, 537-3 Iguchi, Nasushiobara-shi, Tochigi, 329-2763 Japan; 3 Department of Surgery, Yarita Hospital, 899 Goi, Ichihara-shi, Chiba, 290-0056 Japan

**Keywords:** Magnetic compression anastomosis, Esophageal cancer, Colon interposition, Complete stenosis after esophageal cancer surgery

## Abstract

**Background:**

Esophagostomy is important in the treatment of esophageal cancer. However, esophagectomy has a higher risk of postoperative complications. Treatment for complications is often difficult, and in some cases, oral intake is no longer possible. Recently, magnetic compression anastomosis (MCA) was developed; it is a relatively safe method of anastomosis that does not require surgery in patients with stricture, obstruction, or dehiscence of the anastomosis after surgery.

**Case presentation:**

The patient was a 76-year-old Japanese man. He underwent esophagectomy with a three-field dissection for esophageal cancer. A cervical esophagostomy and chest drainage were performed for necrosis of the gastric tube. Following infection control, colon interposition was performed. However, after the operation, the colon necrotized and formed an abscess. Drainage controlled the infection, but the colon was completely obstructed. The patient was referred to our hospital to restore oral ingestion. Contrast studies showed that the length of the occlusion was 10 mm. The reconstruction was examined; reanastomosis by surgery was judged to be a high risk, so the strategy of anastomosis by MCA was adopted. In the operation, the anterior chest was opened to expose the colon, and a magnet was inserted directly into the blind end of the colon. The magnet was guided to the blind end of the esophagus using an oral endoscope. Two weeks after MCA, a contrast study confirmed the passage of the contrast agent from the esophagus to the colon. The patient eventually took 18 bougies after the MCA. However, since then, he has not needed a bougie. As of 1 year and 8 months after the MCA, the patient is living at home with oral intake restored.

**Conclusions:**

MCA is an effective and safe treatment for complete stenosis after esophageal cancer surgery.

## Introduction

Esophagostomy is important in the treatment of esophageal cancer. However, esophagectomy has a higher risk of postoperative complications, which affects recurrence, mortality, costs, and long-term quality of life [1–3].

Currently, colon interposition is the method mainly used for esophageal reconstruction in patients who cannot use the stomach. According to Japanese data, colon interposition was performed in 3.0% of all esophageal cancer patients [4]. Colon interposition is a complex operation with specific indications. Results after colon reconstruction have indicated 0–16% mortality, 0–10% graft necrosis, and 0–15% anastomotic leakage [5]. Treatment for complications is often difficult, and in some cases, oral intake is no longer possible.

Recently, magnetic compression anastomosis (MCA) was developed; it is a relatively safe method of anastomosis that does not require surgery in patients with stricture, obstruction, or dehiscence of the anastomosis after surgery [6–8].

We herein report a patient with complete stenosis and preservation treatment for necrosis after colon interposition that was successfully treated with MCA.

## Case presentation

The patient was a 76-year-old Japanese man. He underwent subtotal esophagectomy through right thoracotomy, with 3-field lymph node dissection, and retrosternal route gastric tube reconstruction, with a diagnosis of thoracic esophageal cancer. On day 21 after the operation, a cervical esophagostomy and chest drainage were performed for necrosis of the gastric tube. Following infection control, esophageal reconstruction of the ante-sternal route via the left colon was performed 63 days after the first operation. On day 71 after the first operation, the anterior wall of the colon near the anastomosis was necrotic about 2 cm and formed an abscess. The neck wound was opened, and drainage was performed. Drainage controlled the infection, but the colon with abscesses was completely stenotic. After controlling the infection, the patient was referred to our hospital (160 days after the first surgery) to restore oral ingestion.

**Fig. 1:**
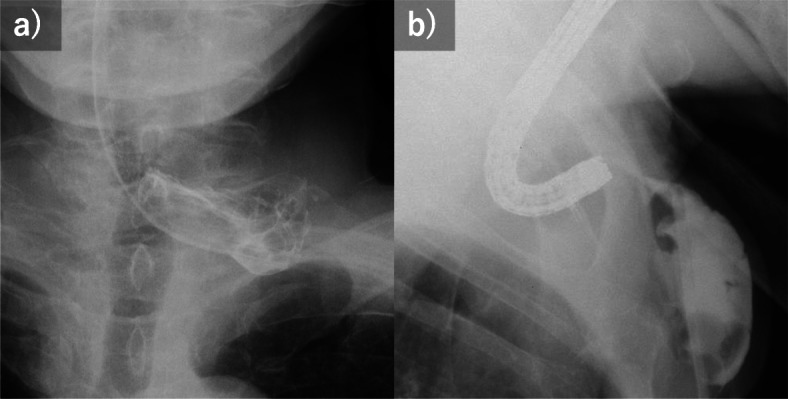
**a** Contrast examination revealed that the esophagus side had a blind end. **b** An endoscope was inserted into the esophagus, and imaging was performed using a catheter placed in the colon. The esophagus side and the colon side were 10 mm apart

**Fig. 2:**
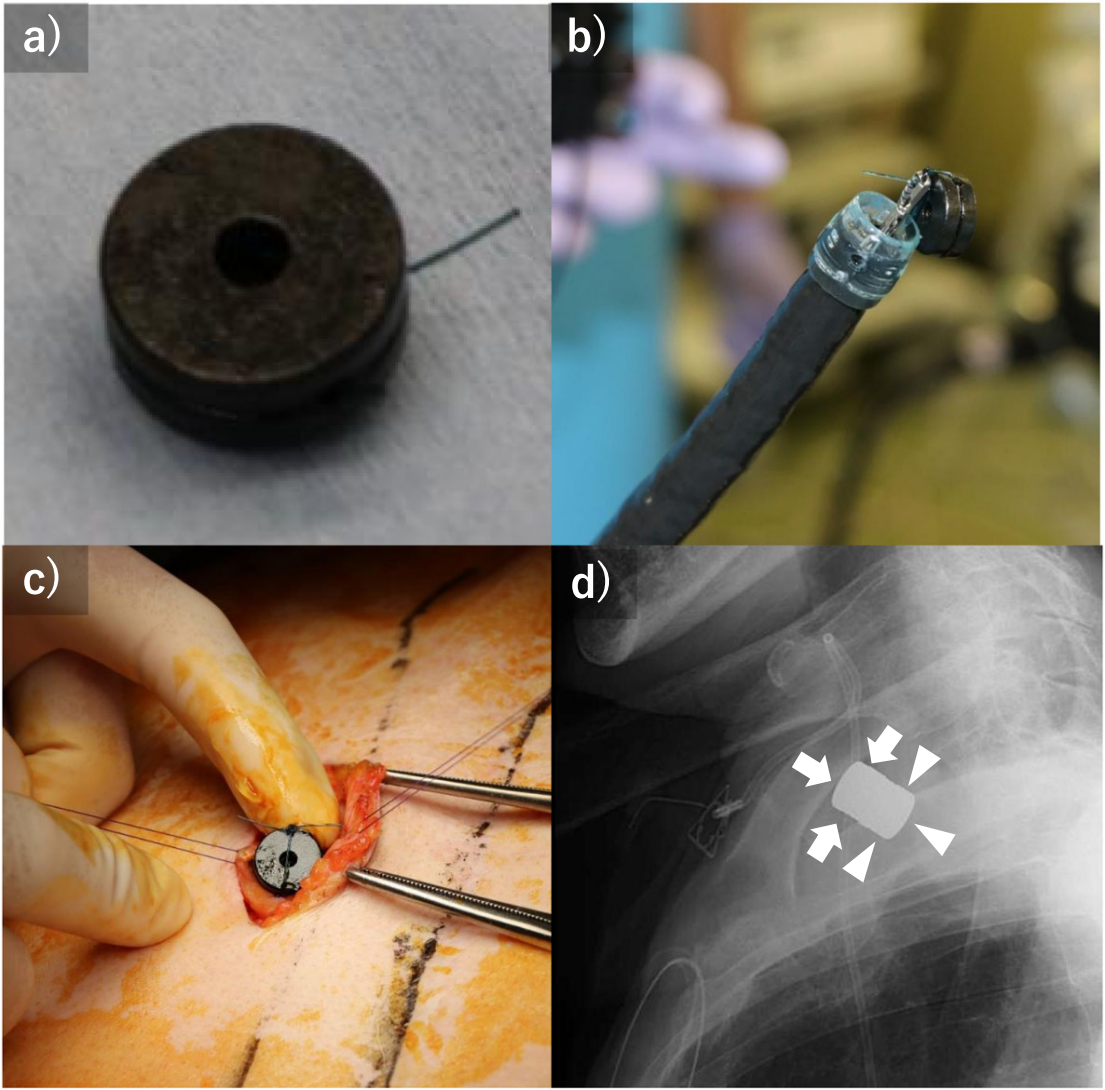
**a** A magnet of 12.5 mm in diameter, 5 mm in thickness, and 3500 gauss was used. **b** A magnet was placed on the left esophagus side using an endoscope. **c** The magnet was inserted directly into the blind end of the colon. **d** It was confirmed by X-ray examination that the magnets left in place were attracted (arrows are the magnets of the left esophagus side; arrow heads are the magnets of the colon side)

**Fig. 3:**
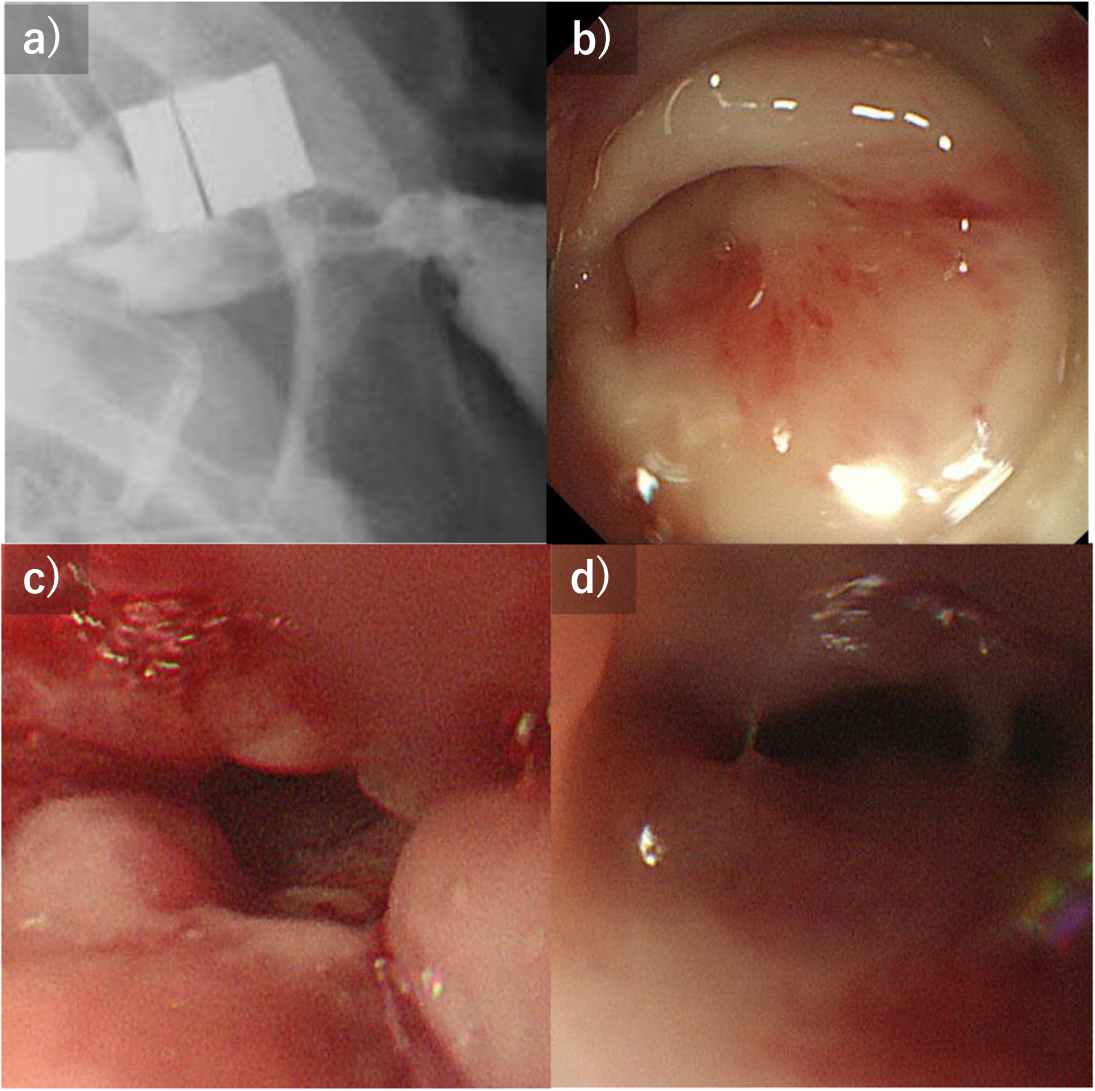
**a** Two weeks after the MCA, a contrast examination was performed, confirming the outflow of the contrast agent form the esophagus to the colon. **b** The endoscopic examination 2 weeks after MC revealed that the lumen was about 5 mm. **c** Endoscopy 1 month after MCA. The bougie is repeating. **d** Endoscopy 6 months after MCA. After a total of 18 bougies, no more bougies were needed

Contrast examination at our hospital revealed that the esophagus had a blind end, and the obstruction distance between the esophagus and the colon was 10 mm (Fig. 1). The reconstruction was examined, reanastomosis by surgery was judged to be a high risk, so the strategy of anastomosis by MCA was adopted. In Japan, MCA was not covered by insurance, so we had many discussions to make preparations. We consulted the ethics review committee of the hospital, referencing past reports, and also obtained consent from the patient after sufficiently explaining this treatment. In addition, we asked an experienced doctor to join us for the actual MCA procedure. Thirteen months after the first operation, an anastomosis between the esophagus and the colon was performed by MCA (Fig. 2). In the operation, the anterior chest was opened to expose the colon, and a magnet (12.5 mm in diameter, 5 mm in thickness, and 3500 Gausses. This magnet is not a commercial product and was developed by Dr. Yamanouchi.) was inserted directly into the blind end of the colon. The magnet was guided to the blind end of the esophagus using an oral endoscope. X-ray examination confirmed that the magnets placed on the oral side and the anal side were close to each other. Two weeks after MCA, a contrast examination confirmed the passage of the contrast agent from the esophagus to the colon (Fig. 3a). The magnet was collected using an endoscope. At this time, the lumen of the anastomotic hole was 5 mm (Fig. 3b). The patient had repeated endoscopic balloon bougies and was discharged 82 days after MCA. Eventually, he had 18 bougies and then they were no longer needed 6 months after the MCA (Fig. 3c, d). As of 1 year and 8 months after the MCA, the patient is living at home with oral intake restored.

## Discussion

Recently, colon interposition has had a limited indication when the stomach cannot be used. According to a recent report, colon interposition was considered to have a lower incidence of regurgitation, aspiration pneumonia, and anastomosis site stricture in long-term survivors [9, 10]. However, 11% of patients undergoing colon interposition underwent reoperation, and there was no significant difference in the median BMI change, median weight loss, or other alimentary functions. Gastric reconstruction is the first choice for esophageal cancer surgery because colon interposition is a complex and nonstandardized procedure.

However, colon interposition plays an important role in esophageal surgery because some patients cannot use the stomach. Colon interposition for salvage treatment also has an important role in esophageal surgery. Reslinger et al reported that colon interposition for salvage treatment was successful compared with its use as primary esophageal surgery [11].

MCA is a treatment technique in which a strong permanent magnet is placed in the segment of the intestinal tract to be anastomosed to form an anastomosis. MCA was invented by Dr. Yamanouchi in the 1998 [8]. In a recent MCA report, enteroenterostomy [12], choledochoenterostomy [8, 9], and duct-to-duct anastomosis [13] have been reported with good results.

To our knowledge, only 9 cases, including our case, of MCA for enteroenterostomy have been reported (Table 1). This is the first report of treatment with MCA for complete stenosis due to abscess formation after esophageal cancer surgery, as in our case. The median age of the patients was 59 years old (range 1–89 years old). MCA has been performed on children and elderly patients, and the approach has been performing well. The median time to remove the magnet was 10 days (range 4–15 days). According to the report, the length of the time period before removal of the magnets depends upon the distance between the two magnets and their strength. The length of time required for anastomosis by the MCA is 7–10 days with choledochoenterostomy and 10 or more days with enteroenterostomy [7]. An additional bougie was administered after MCA in three cases, including our own. According to the report, more than 80% of gastroduodenal anastomosis by MCA requires stenosis and balloon bougie [12]. Clinical trials have reported stent placement after MCA anastomosis for post-MCA stenosis [14, 15].

**Table 1:** Characteristics and outcomes of patients who underwent magnetic compression anastomosis for enteroenterostomy

No.	Author	Age (year)	Sex	Disease	Disease cause	Anastomosis	Time until removal of magnet (days)	Reintervention
1	Robert M Dorman	1	Female	Esophageal atresia	-	Esophagoesophagostomy	13	Balloon dilation
2	Luzia Toselli	4	Male	Ileostomy	Malignant bowel obstruction	Small bowel-small bowel	14	None
		16	Female	Ileostomy	Ileocolonic polyposis	Small bowel-small bowel	15	None
3	Mark Bremholm Ellebaek	1	Male	Esophageal atresia	-	Esophagoesophagostomy	5	Balloon dilation
4	Erkan Parlak	63	Male	Anastomotic obstruction	Larynx carcinoma	Pharyngoesophageal	4	Balloon dilation
5	Carter C. Lebares	59	Male	Small bowel obstruction	-	Small bowel-small bowel	9	None
6	Hideaki Kawabata	72	Male	Anastomotic obstruction	Gastric cancer	Jejunojejunostomy	10	None
7	Hideaki Kawabata	89	Female	Superior mesenteric artery syndrome	-	Gastrojejunostomy	10	None
8	Present case	76	Male	Obstruction due to abscess formation	Esophageal cancer	Esophagostomy	14	Balloon dilation

Advantages of MCA include the simplicity of placing a magnet in the digestive tract and the fact that it is a less invasive approach than surgery. However, as a disadvantage, it can only be performed in limited situations. In cases of stenosis where the lumen is retained, a bougie should be considered as the first choice, as the degree of dilation can be adjusted according to the degree of stricture of the digestive tract, and the technique can be performed multiple times. If the gastrointestinal tract is completely occluded, surgical reanastomosis should be considered first. As mentioned above, the MCA procedure has a drawback in that the ideal conditions for its application are limited. Of note, while such applications are still in the clinical trial stage, MCA can also reportedly be performed for bile duct jejunostomy and pancreaticojejunostomy [16] after pancreaticoduodenectomy. Therefore, despite the associated drawbacks, MCA is expected to be a useful method of performing gastrointestinal anastomosis in the future.

We consider the application of MCA as follows. First, it is necessary to have a method in which magnets can be placed on both sides of the digestive tract as an absolute condition. Second, it is not appropriate in an emergency scenario because it takes time before the anastomosis is performed. After satisfying these conditions, there is an indication for MCA in patients who are judged to have a high risk of surgery because of their general condition, complications, and difficulty in surgical procedures.

In our case, it was expected that it would be difficult to secure the esophagus due to adhesions after surgery and abscess formation and that reflux and aspiration were likely to occur due to the high-level anastomosis. Therefore, anastomosis by MCA was performed instead of anastomosis by surgery.

## Conclusions

We experienced a case in which oral ingestion was possible after MCA for complete stenosis due to abscess formation after esophageal cancer surgery. To our knowledge, this is the first report of treatment with MCA after esophageal cancer surgery. We conclude that MCA is an effective and safe treatment for complete stenosis after esophageal cancer surgery.

## Data Availability

Presented within the manuscript. Please contact author for additional data requests.

## Abbreviations

MCA: Magnetic compression anastomosis
